# Fe-Cr-Nb-B Ferrofluid for Biomedical Applications

**DOI:** 10.3390/nano12091488

**Published:** 2022-04-27

**Authors:** Anca Emanuela Minuti, George Stoian, Dumitru-Daniel Herea, Ecaterina Radu, Nicoleta Lupu, Horia Chiriac

**Affiliations:** 1 Magnetic Materials and Devices Department, National Institute of Research and Development for Technical Physics, 700050 Iasi, Romania; aminuti@phys-iasi.ro (A.E.M.); gstoian@phys-iasi.ro (G.S.); dherea@phys-iasi.ro (D.-D.H.); kathyradu@yahoo.com (E.R.); 2 Faculty of Physics, “Alexandru Ioan Cuza” University, 700506 Iasi, Romania

**Keywords:** ferrofluid, biocompatibility, magneto-mechanical effect, magnetic hyperthermia

## Abstract

A ferrofluid based on Fe_67.2_Cr_12.5_Nb_0.3_B_20_ magnetic particles with a low Curie temperature was prepared. The particles, most of which had dimensions under 60 nm, were dispersed in a calcium gluconate solution, leading to a stable ferrofluid. The obtained ferrofluid had a magnetization of 0.04 to 0.17 emu/cm^3^, depending on the particles’ concentration, and a viscosity that increased nonlinearly with the applied magnetic field. The ferrofluid appeared to be biocompatible, as it showed low cytotoxicity, even at high concentrations and for long intervals of co-incubation with human cells, demonstrating a good potential to be used for cancer therapies through magnetic hyperthermia as well as magneto-mechanical actuation.

## 1. Introduction

Magnetic particles (MPs) are frequently used in biomedical applications due to their specific magnetic properties, the variety of sizes in which they can be obtained, and because they can be tailored to become biocompatible. To direct them through tissues and blood vessels using magnetic fields it is necessary to prevent their agglomeration and sedimentation [1]. This shortcoming can be avoided by using ferrofluids, which are colloidal suspensions of MPs that can be manipulated and controlled by an external magnetic field [2]. The ferrofluids (FFs) usually consist of three basic elements, namely, the ferromagnetic particles in the nanometer range [3], the surfactant that covers the particles in order to prevent their agglomeration, and the liquid carrier which, together with the surfactant, enables the particles to be kept in suspension. The combination of these three components results in a stable colloidal suspension of magnetic nanoparticles (MNPs). Ferrofluids have been investigated for different biomedical applications, such as magnetically guided embolization, magnetic cell separation, drug targeting, magnetic hyperthermia, and magneto-mechanical induction of cancer cell death [4].

Recently, magnetic ferrofluid hyperthermia using biocompatible magnetic nanoparticles as heat mediators has gained more and more attention. Thus, this method has been investigated for cancer therapy because of its high efficacy and limited side effects. Today, the reason for using hyperthermia in the treatment of cancer is well established: the sustained temperatures above 43 °C cause necrosis of cancer cells, which are more sensitive to heat than normal tissues. Thus, hyperthermia is a promising approach for cancer therapy, in part because hyperthermia is a physical treatment that can result in fewer side effects than chemotherapy or radiation therapy [5].

A particularly interesting alternative for the use of ferrofluids is known as cell death induced by magneto-mechanical effect. This technique consists of exerting mechanical forces on the cells using magnetic nanoparticles controlled by low-frequency magnetic fields. This can induce cellular death through apoptosis [6]. Drug delivery using ferrofluids represents another direction that is very much studied at present [7]. Using the magnetic particles from the magnetic fluid as carriers for active substances, a variety of principles can be directed to the targeted area using magnetic fields, thus preventing the absorption of the carried drug in tissues other than the intended ones.

Ferrofluids for biomedical applications are usually based on iron oxides, spinel ferrites, Fe metal, and Fe–Co alloys’ nanoparticles [4]. MNPs are usually prepared by microemulsion, co-precipitation, ball milling, or thermal decomposition methods [3,4]. Iron oxide (Fe_3_O_4_) or cobalt iron oxide (CoFe_2_O_4_) nanoparticles are widely used to prepare FFs for biomedical applications—particularly for hyperthermia—because of their relatively easy processing and good biological compatibility [3,4].

Recently, amorphous nanoparticles were proposed for the preparation of ferrofluids for hyperthermia—mainly for their good magnetic properties. Magnetic Fe-B, Fe-Ni-B, and Co-B amorphous nanoparticles were successfully synthesized via chemical reduction by Yang et al. [8]. The structure, morphology, and magnetic properties of the amorphous nanoparticles, their magneto-viscous properties, and the hyperthermia effects of the corresponding FFs were studied. Amorphous Co-Fe-Si-B magnetic nanoparticles, prepared via chemical reduction, were used by Wang et al. to prepare ferrofluids consisting of particles covered with sodium oleate [9]. Ferrofluids based on amorphous Fe-Co-B magnetic nanoparticles obtained via microemulsion were prepared by Zhao et al. [10], using oleic acid as a surfactant and water or silicone oil as a liquid carrier. Magnetorheological fluids based on amorphous Fe-Si-B particles obtained via milling of amorphous ribbons were prepared by Yu et al. [11], using oleic acid as a surfactant and silicon oil as a liquid carrier. From the literature data [8,9,10,11], we can conclude that saturation magnetization varies between 40 and 75 emu/g, while the Curie temperature ranges between 300 °C and 500 °C, for the reported amorphous particles used to prepare the ferrofluids.

Recently, we developed a new type of amorphous magnetic particle based on glassy Fe_79.7−x_Cr_x_Nb_0.3_B_20_ alloys (x = 11.5 ÷ 13 at.%) [12]. The nanoparticles are prepared by mechanical milling of precursor amorphous melt-spun ribbons obtained via rapid solidification. They have sizes between 5 nm and 5 µm, rectangular shapes, and very good magnetic properties, such as high saturation magnetization, *M_s_*, between 40 and 80 emu/g, low Curie temperature (*T_C_* varies between 16 and 50 °C, depending on Cr content), and high magnetic anisotropy, *K*, caused by their shape anisotropy and the superferromagnetic behavior of the precursor amorphous magnetic ribbons. These characteristics make Fe-Cr-Nb-B magnetic particles particularly attractive for cancer therapy through hyperthermia, magneto-mechanical effects, and drug delivery [12,13]. The interaction between magnetic particles and the cell culture medium is facilitated if the particles are delivered through a supporting liquid medium. In this sense, the use of a ferrofluid based on these particles is very appropriate. The particles appear to be suitable for self-regulated hyperthermia [14] and for the destruction of cancer cells by magnetomechanical effects [13].

The purpose of the present work was to prepare a ferrofluid based on a suspension of low-Curie-temperature Fe_67.2_Cr_12.5_Nb_0.3_B_20_ magnetic particles with good stability that prevents their agglomeration—aspects that are important in biomedical applications. The methodologies for obtaining Fe-Cr-Nb-B particles and for using them for magnetic hyperthermia are described in detail elsewhere [12,14]. Moreover, we have previously reported details about the in vitro testing of these particles for the destruction of cancer cells through magneto-mechanical effects [13].

## 2. Materials and Methods

MPs were obtained via high-energy ball milling of precursor Fe_67.2_Cr_12.5_Nb_0.3_B_20_ amorphous melt-spun ribbons in sodium oleate/oleic acid, using a Retsch 200 high-energy ball mill. An estimation of the size distribution along with the information about the dimensions of the particles was obtained using a NEON 40EsB CrossBeam ultra-high-resolution scanning electron microscope (UHR-SEM) from Carl Zeiss GmbH, München, Germany, equipped with an energy-dispersive X-ray spectroscopy (EDS) module for compositional studies, and with a focused ion beam for the study of layers covering the MPs. The size distribution of the particles was obtained from the SEM images, using specialized software. For the structural characterization of the particles, we used a Bruker AXS D8-Advance diffractometer from Bruker GmbH, Bremen, Germany.

The heating efficiency of ball-milled Fe-Cr-Nb-B powders was investigated in an a.c. field of 3500 Oe (f = 153 kHz) using a homemade magnetic-induction hyperthermia unit, consisting of an HF generator (HFG3 IGBT from Eldec Induction GmbH, Dornstetten, Germany) and a specially designed temperature measurement system adapted to HF working conditions (Optical Thermometer from Optocon AG, Germany) [12]. In order to check the effect of magnetic particles’ movement on the viability of cells, we designed a system consisting of four coils placed in cross that could produce a rotating magnetic field. We designed a special electronic system allowing us to set the magnetic field’s intensity, its frequency, and the time of exposure [13].

For preparing the ferrofluid, the obtained particles were dispersed with a UP50H ultrasonic probe from Hielscher Ultrasonic GmbH, Teltow, Germany, by using a calcium gluconate solution (94 mg/mL; pH = 6 ÷ 7) as a dispersing medium.

The magnetic properties of the MPs and those of the ferrofluid were investigated using a 7410 Series Vibrating Sample Magnetometer (VSM) from Lake Shore Cryotronics, Inc., Westerville, OH, USA. The temperature dependence of the magnetization was evaluated with the same VSM equipment, at a heating rate of 150 °C/min, in the temperature range 20–80 °C. The rheological modifications in the presence of an applied magnetic field were studied using a Physica MCR 101 rheometer from Anton Paar GmbH, Ostfildern, Germany. For ferrofluid evaluation, the particle suspensions were sonicated with an ultrasonic homogenizer working in pulses (UP50H from Hielscher Ultrasonic GmbH, Teltow, Germany), at 90% amplitude.

Biocompatibility tests were conducted on the human osteosarcoma cell line (HOS) MG-63 from Sigma-Aldrich, as well as on normal human dermal fibroblasts (NHDFs). Evaluation of the presence of magnetic particles inside cells was performed using a protocol described by Schrand et al. [15]. For magnetic hyperthermia and magneto-mechanical actuation, 5 × 10^4^ cells were seeded in 24-well plates and grown until 85 ÷ 90% confluency. The complete cell culture medium was replaced with the ferrofluid dispersed in the cell culture medium, at a concentration of 2 mg/mL of MPs, which was later added to the cells and co-incubated for another 24 h. Following this period, the samples were either treated in an alternative magnetic field with a frequency of 153 kHz and a magnetic field of 3500 Oe for 60 min., for magnetic hyperthermia, or they were treated twice—once every 24 h, for 30 min., in a rotating magnetic field of 80 Oe at a frequency of 3 Hz for magneto-mechanical actuation.

## 3. Results

### 3.1. Fe_67.2_Cr_12.5_Nb_0.3_B_20_ Magnetic Particles

**Figure 1:**
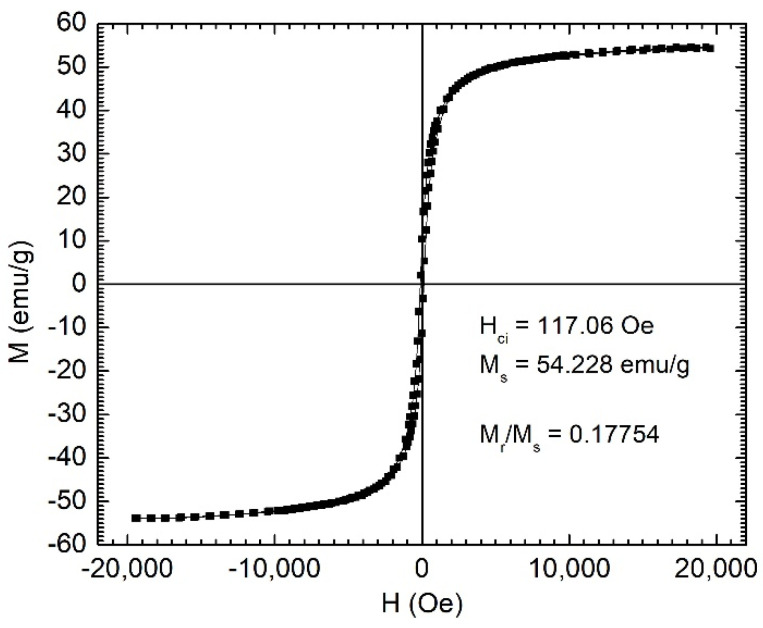
Magnetic hysteresis loop for Fe_67.2_Cr_12.5_Nb_0.3_B_20_ MPs. The measurement was performed at room temperature.

The preparation process included obtaining the melt-spun amorphous ribbons via rapid quenching of the melt on a rotating metallic disk, their annealing for embrittlement, followed by grinding in a high-energy mill in oleic acid or sodium oleate. Our previous studies showed that the variation of Cr content in the alloy influences the equilibrium temperature, which is strongly correlated with the Curie temperature of the MPs. The magnetization curves of the MPs indicate saturation magnetizations of 54 emu/g, as shown in Figure 1. The specific magnetic characteristics of the ribbons obtained by rapid solidification, including the low Curie temperature, are related strongly to the presence of the amorphous phase [16]. Thus, it is extremely important to preserve the initial amorphous state in the resulting powders produced by milling the precursor melt-spun ribbons. This is achievable by a very rigorous control of the milling conditions, including the use of some specific surfactants [12,13,14].

**Figure 2:**
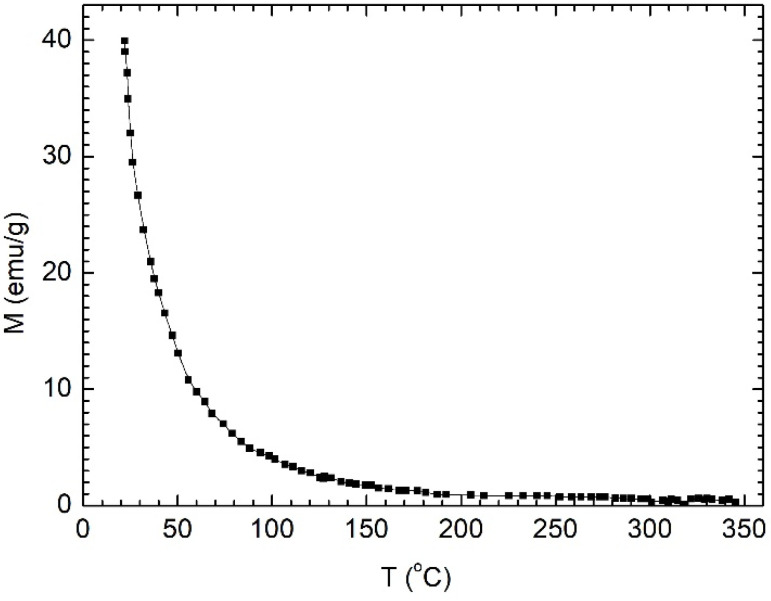
The variation of saturation magnetization with temperature for Fe_67.2_Cr_12.5_Nb_0.3_B_20_ MPs indicates a *T_C_* ≈ 47 °C.

**Figure 3:**
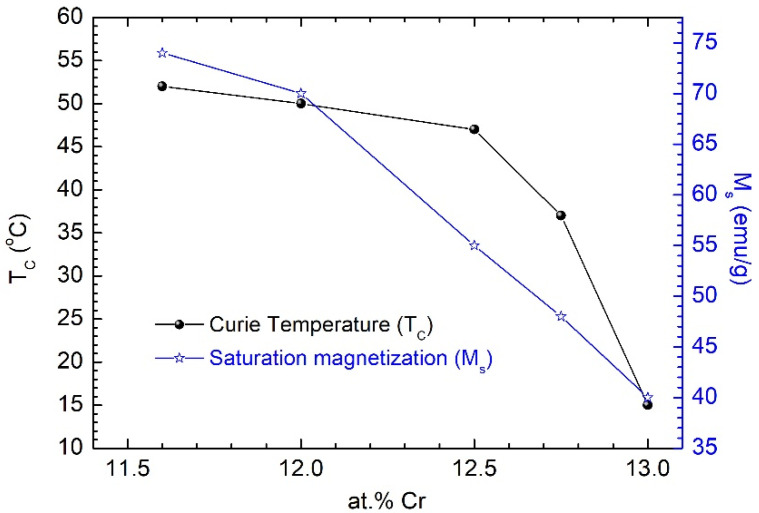
Dependence of the Curie temperature (*T_C_*) and the saturation magnetization (*M_s_*) of the particles on the Cr content of the alloy.

The temperature dependence of the saturation magnetization for Fe_67.2_Cr_12.5_Nb_0.3_B_20_ MPs is presented in Figure 2 and indicates a Curie temperature (*T_C_*) of around 47 °C, which makes them very suitable for magnetic hyperthermia, among other uses. The Curie temperature was determined by the line projection method [17]. The dependence of *T_C_* and *M_s_* on Cr content for the studied MPs is presented in Figure 3. For applications in self-controlled hyperthermia, we used particles with a specific Cr content of 12.5% and a Curie temperature of 47 °C.

**Figure 4:**
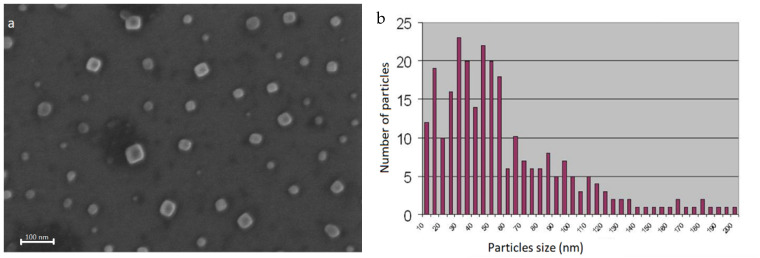
(**a**) UHR-SEM image of the MPs; (**b**) size distribution of MPs.

UHR-SEM images (Figure 4) show that the majority of particles had sizes below 60 nm, making them suitable for ferrofluid preparation, as well as for biomedical applications [18].

### 3.2. Fe_67.2_Cr_12.5_Nb_0.3_B_20_ MP-Based Ferrofluids

The ferrofluids consist of magnetic particles, a surfactant, and the carrier liquid that works with the surfactant to suspend the magnetic particles. We used amorphous Fe_67.2_Cr_12.5_Nb_0.3_B_20_ MPs that were repeatedly washed with a hot solution of NaOH 5% to remove the excess of sodium oleate from the milling process, and then rinsed them several times with water to remove NaOH. A thin layer of sodium oleate still remained on the MPs, acting as a surfactant. The thickness of this layer was around 6–8 nm, as measured by UHR-SEM with a focused ion beam.

We tested different types of carrier liquid to prepare a stable ferrofluid, such as sodium oleate [19], citric acid [20], and calcium gluconate, and we varied the parameters involved in the reaction. Calcium gluconate proved to be the best option for dispersing and stabilizing this type of particle.

**Figure 5:**
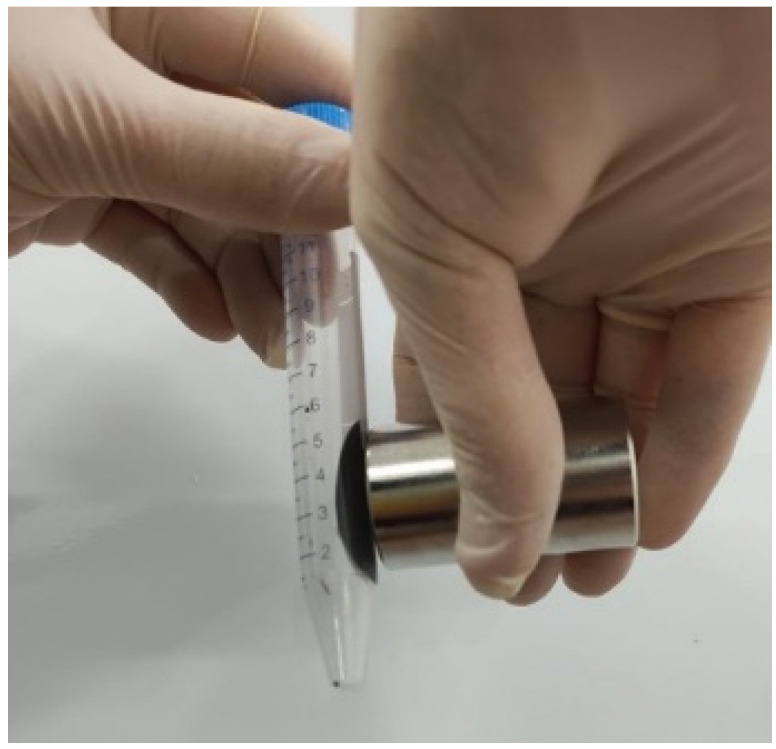
Ferrofluid behavior in the presence of a magnet.

After washing, the particles were put in contact with a calcium gluconate solution, which acted as a dispersing agent. The mixture was ultrasonicated for 30 min at 80 °C, and then allowed to settle for 12 h. The ferrofluid’s behavior in the presence of a magnet is presented in Figure 5.

**Figure 6:**
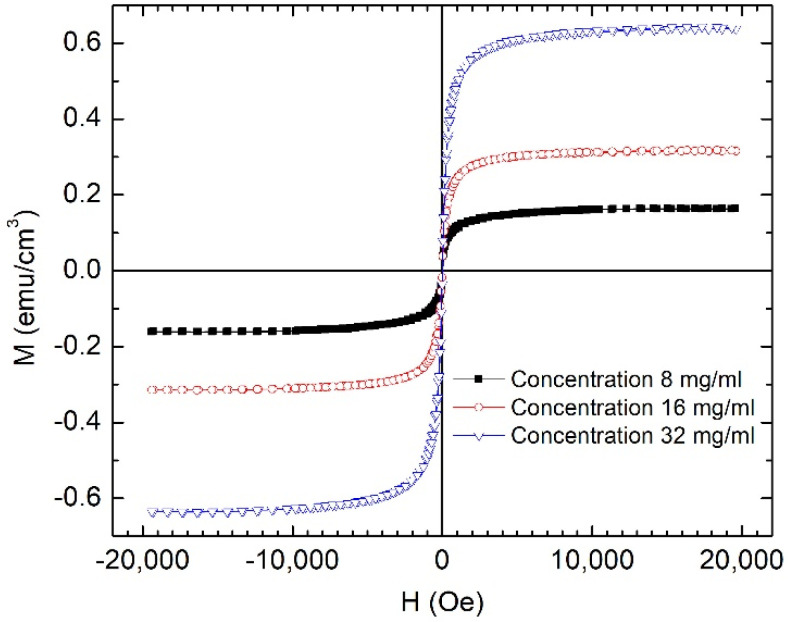
Magnetic hysteresis loops for Fe_67.2_Cr_12.5_Nb_0.3_B_20_ MP-based ferrofluids with 3 different concentrations of MPs in calcium gluconate.

The anhysteretic behavior of the magnetization curves (shown in Figure 6) emphasizes the high colloidal stability of the obtained ferrofluid.

An indication of the stability of the magnetic fluid can be obtained by observing the sedimentation ratio. The sedimentation ratio is calculated according to the following formula:

$$\mathrm{Sedimentation}\;\mathrm{ratio}\;\left(\%\right)=\frac{\mathrm{Supernatant}\;\mathrm{volume}}{\mathrm{Total}\;\mathrm{suspension}\;\mathrm{volume}}\times 100\%,$$

where the supernatant is the amount of clear fluid (calcium gluconate) separated from the suspension and located above the particle suspension.

**Figure 7:**
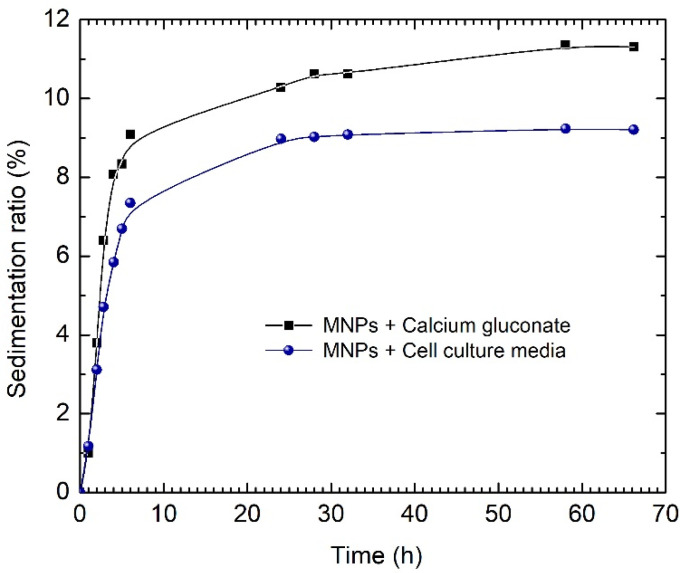
Sedimentation ratio of an Fe-Cr-Nb-B MP-based ferrofluid in calcium gluconate and cell culture medium.

To assess the colloidal stability of the ferrofluid based on calcium gluconate, we evaluated the sedimentation ratio in calcium gluconate and in cell culture medium for 60 h. As one can see from Figure 7, the culture medium decreased the sedimentation ratio in comparison with calcium gluconate. The enhanced stability of the ferrofluid based on calcium gluconate was mostly due to the presence of the proteins in the fetal bovine serum (FBS)—the main component of the culture medium. As is well documented, these proteins stick around the particles and form a so-called “protein corona”, leading to better dispersion of the magnetic particles and, thus, well preventing the particle agglomeration caused by steric repulsions. Consequently, the critical stability test fundamentally concerns the ferrofluid as it was prepared. Figure 7 shows an accelerated sedimentation of the ferrofluid in the first 10 h. However, the sedimentation ratio remains below 10%, and further tends to display a steady-state behavior for the remaining time period of 55 h, indicating a proper stability value for this time period.

Magnetorheological measurements on the magnetic liquid determined the dynamic viscosity of the fluid in the magnetic field and in its absence. The measuring system used parallel plates placed at a distance of 0.25 mm and discs with a diameter of 20 mm. The temperature was kept constant at 26 °C during the measurements, with the help of a Paar Physica Viscotherm VT2 thermostat.

**Figure 8:**
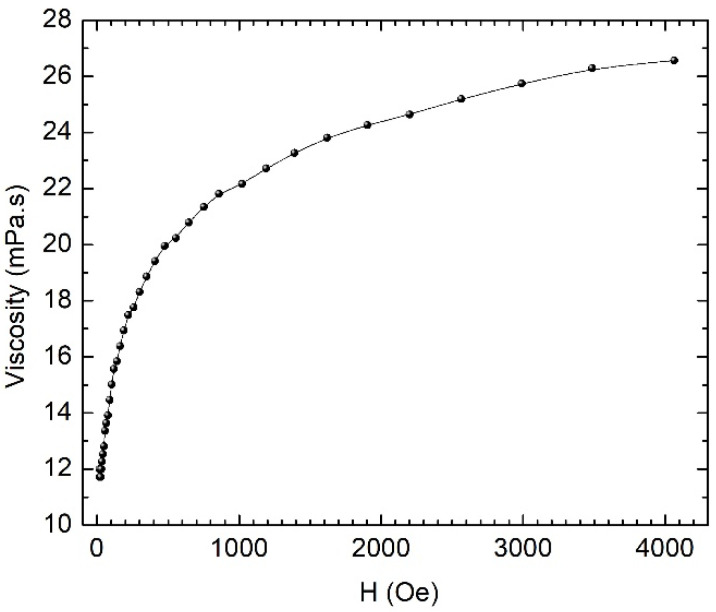
The dependence of the viscosity of the ferrofluid on the applied magnetic field.

For our studies, we chose the fluid with the concentration of 8 mg/mL and measured the dependence of the viscosity on the magnetic field. This dependency is shown in Figure 8. The viscosity measured outside the magnetic field was approximately 12 × 10^−3^ Pa.s (12 cP)—a value comparable to that of the human blood, which is approximately 5 × 10^−3^ Pa.s (5 cP). The magnetorheological measurements were performed at a share rate of 25 s^−1^. In the magnetic field, the viscosity of the fluid increased approximately 2.2 times.

There was a small magnetorheological effect, which means that the magnetic liquid behaves largely like a ferrofluid. The flow properties of a magnetic fluid/ferrofluid in a magnetic field are determined by the viscous dissipation phenomena related to the movement of both individual particles and particle aggregates in the dispersion medium, leading to the magneto-viscous effect. The viscous dissipation phenomenon is an irreversible process, by means of which the effect of a fluid on adjacent layers, due to the action of shear forces, is transformed into heat. In a uniform magnetic field, the particles’ rotation is prevented by the magnetic field, leading to an increase in viscosity. The changes induced by the magnetic field in the flow behavior of ferrofluids are usually not significant, showing only a reduced magneto-viscous effect, and not developing a yield stress like in magnetorheological fluids. For the latter, the viscosity dramatically increases by up to two orders of magnitude, due to particles’ aggregation (particle chains) in diluted magnetic fluids/ferrofluids. Without agglomerates, with only individual dipolar magnetic particles suspended in the base fluid, the increase in viscosity with the magnetic field is the result of the viscous dissipation that occurs during the relative movement between the particle and the dispersion medium.

The measured values of the viscosity of the ferrofluid in the absence of the magnetic field fall within the appropriate range for use in hyperthermia and magneto-mechanical effects. The increase in the viscosity with the field, which certifies the formation of the ferrofluid, does not affect its use in hyperthermia or in magneto-mechanical effects, because the used magnetic fields do not lead to significant increases in the viscosity.

**Figure 9:**
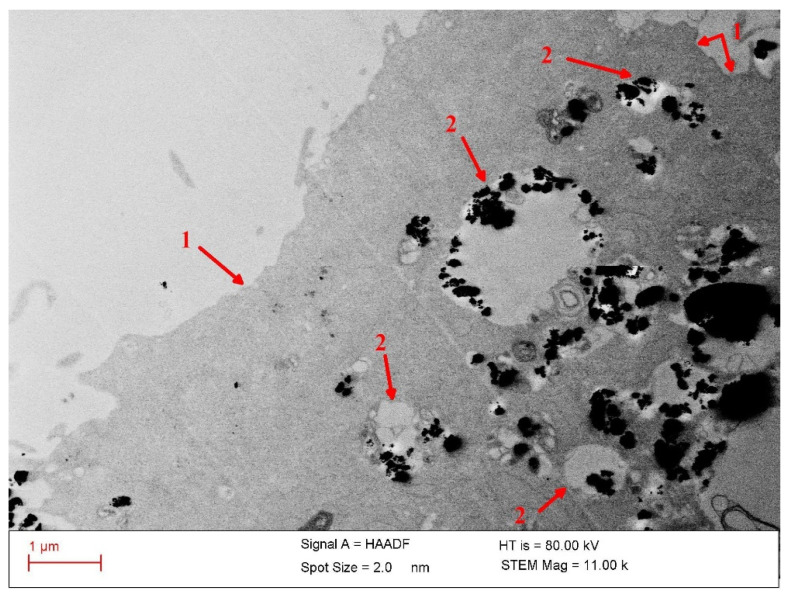
STEM image of the cross-section of an osteosarcoma cell with Fe_67.2_Cr_12.5_Nb_0.3_B_20_ magnetic particles after 24 h of co-incubation. Arrows labelled “1” indicate the cell’s membrane, while arrows labelled “2” indicate the lysosomes with magnetic particles.

The colloidal stability of the particles in culture media is validated by the TEM observations, which show a fine distribution of the nanoparticles internalized in cells (Figure 9). This indicates a good prior dispersion of the particles in the Dulbecco cell culture medium supplemented with fetal bovine serum (FBS) during incubation with cells.

### 3.3. Evaluation of the Biocompatibility of Calcium Gluconate–Fe_67.2_Cr_12.5_Nb_0.3_B_20_ MP-Based Ferrofluids on HOS Cells and NHDFs

The MPs showed good biocompatibility and a low tumoricidal effect, as our previous studies depicted [14]. When sodium oleate was chosen for the milling process and calcium gluconate as a dispersing agent, we also considered their biocompatibility. Sodium oleate is known to be biocompatible [21], and it is useful for preventing the agglomeration of particles. Calcium gluconate solution is widely used for hypocalcemia treatment and has a wide range of biomedical applications. All the components of the ferrofluid are biocompatible if they are used by themselves, but it is important to appreciate their cytotoxicity levels when used together.

To study the cytotoxicity of the ferrofluid, 10^4^ HOS cells and NHDFs were seeded in a 96-well Corning Costar cell culture plate and allowed to reach confluence at 37 °C and 5% CO_2_. When the plate reached 85–90% confluence, the complete cell culture medium with 10% fetal bovine serum (Dulbecco’s Modified Eagle Medium, Life Technologies, San Diego, CA, USA) was replaced with two different concentrations (2 and 5 mg/mL, respectively) of ferrofluid dispersed in the cell culture medium, which was later added to the cells and incubated for another 24 h. After the incubation period, the MTT assay was conducted following the manufacturer’s indications.

The cellular viability of the sample (%) was calculated using the following equation:

$$\mathrm{CV}\;(\%)=100\times \frac{{\mathrm{OD}}_{\mathrm{Ferrofluid}}-{\mathrm{OD}}_{\mathrm{Blank}}}{{\mathrm{OD}}_{\mathrm{Control}}-{\mathrm{OD}}_{\mathrm{Blank}}},$$

where CV (%) represents the cellular viability and OD represents the optical density of the wells containing (a) cells with ferrofluid (OD_Ferrofluid_), (b) cells only (OD_Control_), and (c) cell culture medium without cells (OD_Blank_). The absorbance of the samples was measured at 570 nm using a Synergy HTX Multi-Mode Microplate Reader from BioTek Instruments (now part of Agilent Technologies), Santa Clara, CA, USA.

**Figure 10:**
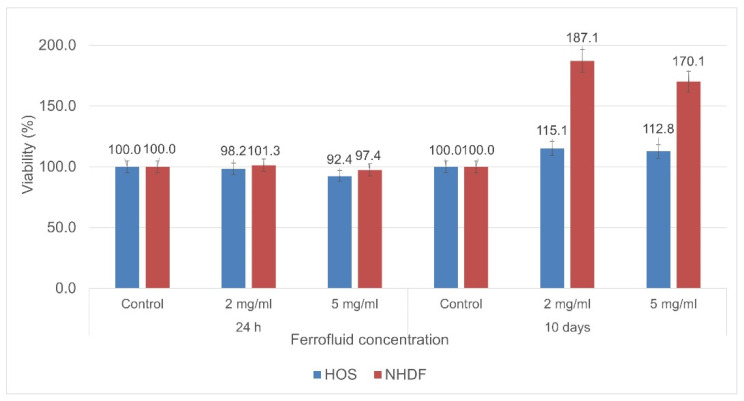
Cell viability of HOS cells and NHDFs after 24 h and 10 days of cell–ferrofluid co-incubation.

The ferrofluid showed excellent biocompatibility, both in the short and long term (10 days), when tested with NHDFs and HOS cells. The cytotoxicity results highlighted that the cell viability slightly decreased for the samples evaluated 24 h after co-incubation—especially the ones with a higher concentration of particles. However, it remained at high levels, with the calcium gluconate ferrofluid showing a high biocompatibility (Figure 10). In the case of the samples evaluated after 10 days, their viability was higher than that of the control samples, proving that the evaluated particles have no cytotoxic effects on cells even if they are co-incubated for such a long period of time. Furthermore, the results highlight that the presence of these particles leads to high metabolic activity and proliferation—particularly for NHDFs.

The quantitative measurement of the cells’ metabolic processes represents one of the most utilized and reliable methods to evaluate the cell viability. Generally, such measurements are performed after 2–3 days of cell incubation. However, in order to have a strong indication of the cell viability after continuous long-term exposure to magnetic particles, we expanded the time window of the viability experiments to 10 days of incubation. This approach gave us reliable data regarding the cytotoxicity of the magnetic particles; thus, we can appreciate that phenomena such as apoptosis are not induced by the sole presence of the ferrofluid in the cell culture medium.

### 3.4. Destruction of Cancer Cells 

The magnetic behavior of Fe-Cr-Nb-B MPs depends essentially on the Cr content, as well as on the milling conditions of the precursor glassy melt-spun ribbons. A change in the Cr content from 11.6 to 13 at.% changes the Curie temperature from 52 °C to 15 °C. The decrease in the Cr content in the alloy leads to an increase in the saturation magnetization of the alloy [12]. Different biomedical applications of Fe-Cr-Nb-B MPs involve alloys with different Cr contents. Thus, for hyperthermia, alloys with Cr contents between 12 at.% and 12.5 at.% are indicated, while for applications using magneto-mechanical effects involving the rotation of particles in a rotating magnetic field, a lower Cr concentration is preferable (11.5 at.%), but either variant is compatible. We found that the formation of a ferrofluid based on these alloys is independent of the Cr content within the limit of variation considered. Fe-Cr-Nb-B MPs were successfully used for in vivo and in vitro experiments [12,13,14] to evaluate their applicability for the destruction of cancer cells through magnetic hyperthermia or magneto-mechanical effects. The magnetic particles from the ferrofluid injected in the cancer area are ingested by the cancer cells, followed by the application of a magnetic field that induces a higher temperature in the particles which, therefore, heat and affect the cancer cells.

The basis of the magneto-mechanical actuation in cells is the spatial rotation of the magnetic nanoparticles when an external rotating magnetic field is applied. Consequently, the field produces a magnetic torque that depends on the characteristics of the applied magnetic field, as well as on the magnetic moment and the magnetic susceptibility of the nanoparticles. The rotation of the magnetic particles with shape anisotropy leads to the destruction of the cancer cells, which confers superior properties for medical applications to these ferrofluids based on glassy magnetic particles.

**Figure 11:**
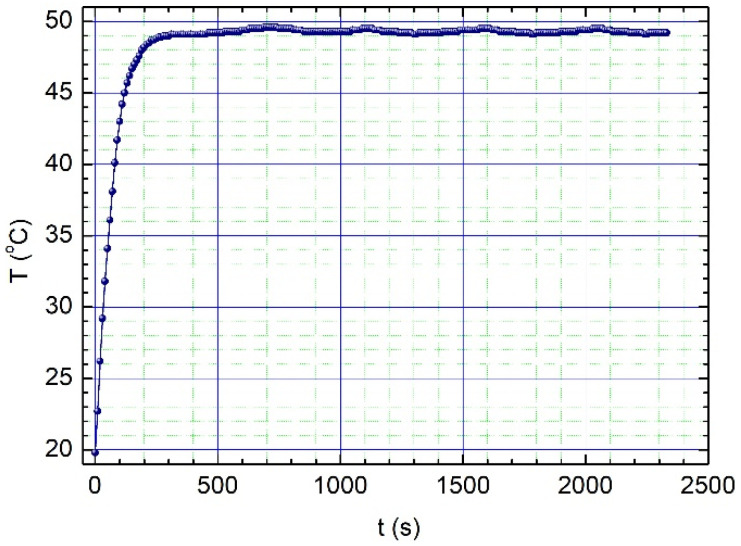
The heating efficiency curves of Fe-Cr-Nb-B MPs vs. time. The tests were conducted in an a.c. field of 3500 Oe (f = 153 kHz) created by a homemade magnetic-induction hyperthermia unit.

The evaluation of the ferrofluid efficiency is presented in Figure 11. After a fast increase from 20 °C to 40 °C in the first 100 s, the temperature follows a steady-state profile, independent of the heating time. This equilibrium temperature is in good agreement with the specific *T_C_* of this type of magnetic particle, i.e., 45 ÷ 47 °C, indicating a good potential for magnetic self-controlled hyperthermia applications. The calculated specific absorption rate value for the studied nanoparticles is 183 W/g.

**Figure 12:**
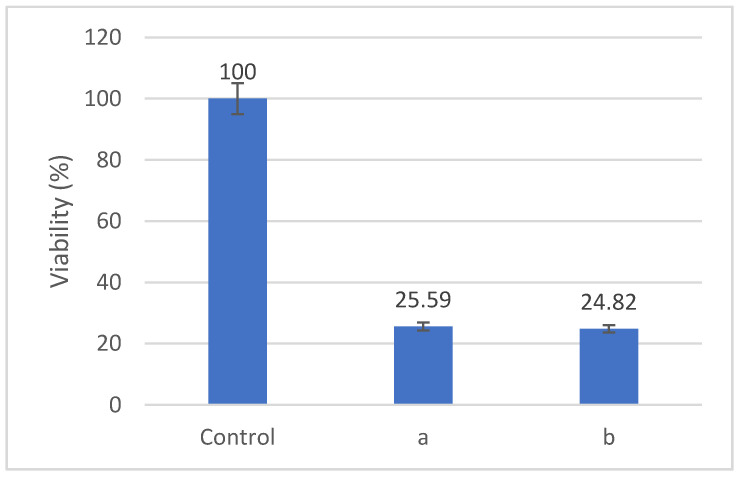
Cell viability after applying (**a**) magnetic hyperthermia and (**b**) magneto-mechanical actuation.

We evaluated the effects of Fe-Cr-Nb-B ferrofluid on the viability of HOS cells after treatments involving either magnetic hyperthermia or magneto-mechanical actuation. Figure 12 shows that the viability of HOS cells decreased to about 25% after 60 min. of treatment with an alternative magnetic field with a frequency of 153 kHz and a magnetic field of 3500 Oe. The samples were co-incubated with 2 mg/mL particle concentration for 24 h, subjected to the alternate magnetic field, and evaluated after a further 24 h. The cell culture medium reached a temperature of approximatively 45 °C during the experiment.

For magneto-mechanical actuation (Figure 12), the samples were also co-incubated with 2 mg/mL of particles for 24 h, followed by field exposure. In this application, the samples were treated twice—once every 24 h—for 30 min. in a rotating magnetic field of 80 Oe at a frequency of 3 Hz. The cell viability was evaluated 24 h after the last MMA treatment, and it decreased by up to 25% following the application of a treatment in a rotating magnetic field. The destructive effects of magnetic hyperthermia or magneto-mechanical actuation on HOS cells are determined by the presence of MPs both outside and inside the cells (Figure 9).

As Figure 9 shows, the significantly increased density of the MPs—internalized mostly into lysosomes after 24 h of co-incubation in cell culture medium—underscores a highly efficient transport of the particles into the tumor cells. The high uptake of the MNPs affords the opportunity for a mixed approach to cancer therapy by applying targeted drug delivery coupled with magnetic hyperthermia and magneto-mechanical effects in order to highly enhance the antitumor efficacy. The cell destruction process is assumed to be induced by apoptosis following the injury of the lysosome membrane and release of the inner digestive enzymes into the cytoplasm.

The efficiency of Fe-Cr-Nb-B NP in killing tumor cells via magneto-mechanical action is shown in a recent comprehensive review in comparison with other types of NP, as already cited in the manuscript [6]. The decrease in tumor cells’ viability to 55% after magneto-mechanical actuation was within the range of reported killing efficiencies, but above the average [6].

As regarding the magnetic hyperthermia, the killing efficiency of Fe-Cr-Nb-B NP (about 50% cell death) [14] was proven to be in the range of the values reported in the literature (13–85%) [22,23]. Nevertheless, it should be emphasized that such a ferrofluid with a low Curie temperature brings an important advantage in the field of self-controlled hyperthermia, as the need for precise temperature control of a target tumor tissue, filled with such a ferrofluid and subjected to specific alternating magnetic fields, is practically eliminated.

## 4. Conclusions

A ferrofluid that becomes stable after the first 24 h, using calcium gluconate as a dispersing agent and low-Curie-temperature Fe_67.2_Cr_12.5_Nb_0.3_B_20_ magnetic particles, was prepared. The saturation magnetization of the ferrofluid is suitable for biomedical applications, and its equilibrium temperature of 47 °C, along with its biocompatibility, makes calcium gluconate–Fe_67.2_Cr_12.5_Nb_0.3_B_20_ MP-based ferrofluids a very good candidate for magnetic hyperthermia applications and for magneto-mechanical destruction of cancer cells. The used procedures allow the preparation of ferrofluids from a wider range of alloys with different Cr contents, depending on the final biomedical application. Using this method, we obtained an improved saturation magnetization for the ferrofluid, a higher concentration of magnetic material in the ferrofluid, and a reduced degree of sedimentation. Furthermore, the obtained ferrofluid is biocompatible, and was confirmed to be ingested by human cells. The newly prepared ferrofluid is also efficient in the destruction of cancer cells both through magnetic hyperthermia and magneto-mechanical effects.
